# Supplementary material for: The interactome of intact mitochondria by cross-linking mass spectrometry provides evidence for coexisting respiratory supercomplexes
Source: Mol Cell Proteomics. 2017 Dec 8;17(2):216–32. doi: 10.1074/mcp.RA117.000470 (PMC5795388; doi:10.1074/mcp.RA117.000470)
Supplement: Supplemental Data [file supp_RA117.000470_133922_0_supp_23978_fzffwf.zip › spectra_annotation/mito_DR_spectra_annotation/312-2-8-1-6-1.pdf]

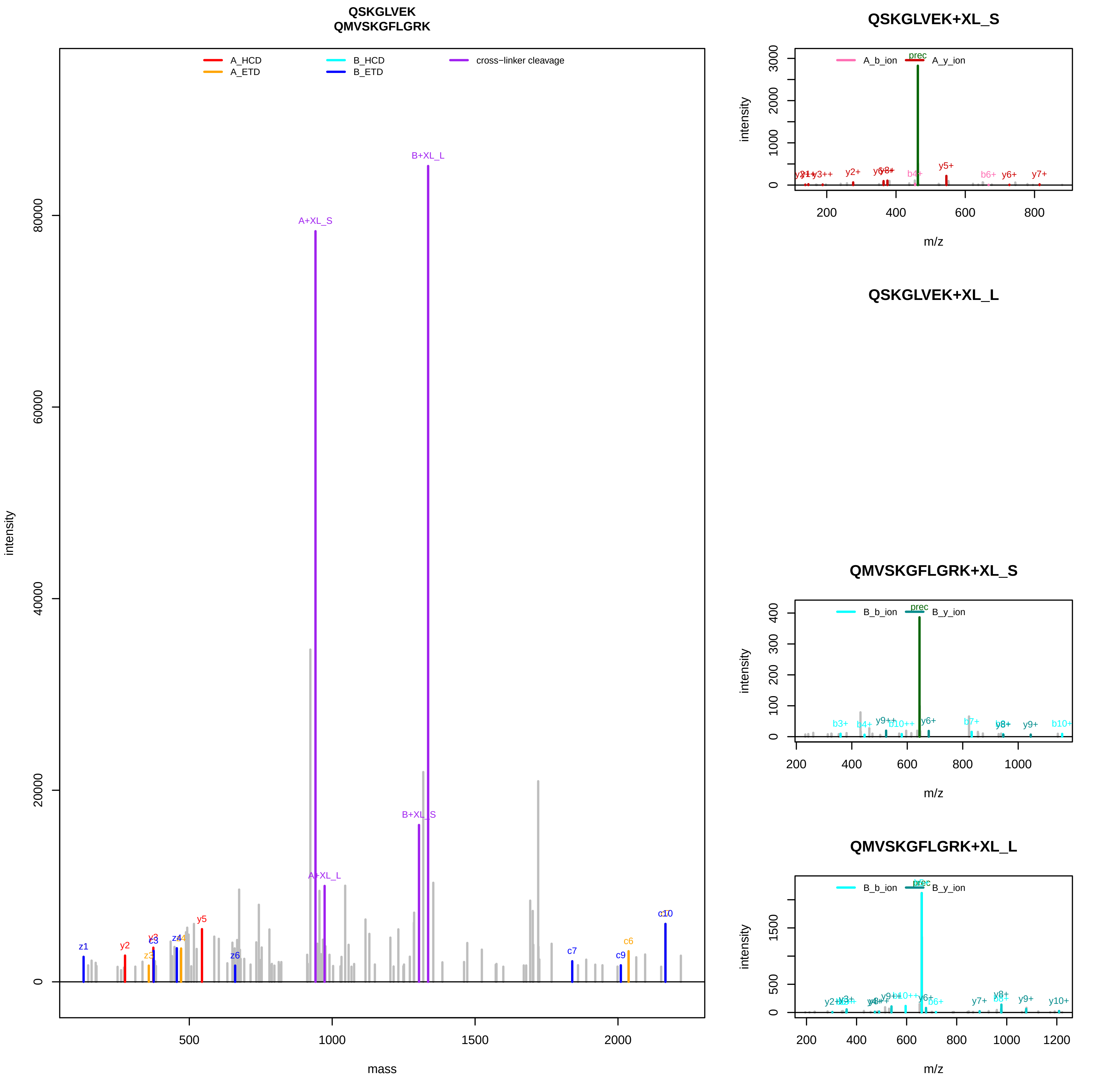

QSKGLVEK
QMVSKGFLGRK
A_HCD
A_ETD
B_HCD
B_ETD
cross−linker cleavage
B+XL_L
A+XL_S
B+XL_S
A+XL_L
intensity
mass
0
20000
40000
60000
80000
500
1000
1500
2000
z1
y2
y3
c3
z3
z4
y5
z6
c7
c9
c6
c10
QSKGLVEK+XL_S
A_b_ion
A_y_ion
prec
y2+
y5+
b4+
b6+
y6+
y7+
m/z
QSKGLVEK+XL_L
QMVSKGFLGRK+XL_S
B_b_ion
B_y_ion
b3+
b4+
y6+
b7+
y8+
y9+
b10+
QMVSKGFLGRK+XL_L
y7+
y9+
y10+
b6+
